# Barriers and Mitigating Strategies to Healthcare Access in Indigenous Communities of Canada: A Narrative Review

**DOI:** 10.3390/healthcare8020112

**Published:** 2020-04-26

**Authors:** Nam Hoang Nguyen, Fatheema B. Subhan, Kienan Williams, Catherine B. Chan

**Affiliations:** 1 Department of Agriculture, Food and Nutritional Sciences, University of Alberta, Edmonton, AB T6G 2E1, Canada; 2 School of Public Health, University of Alberta, Edmonton, AB T6G 1K4, Canada; 3 Population, Public and Indigenous Health Strategic Clinical Network, Alberta Health Services, Calgary, AB T2W 1S7, Canada; 4 Department of Physiology, University of Alberta, Edmonton, AB T6G 2H7, Canada; 5 Diabetes, Obesity and Nutrition Strategic Clinical Network, Alberta Health Services, Edmonton, AB, T2W 1S7, Canada

**Keywords:** healthcare accessibility, Indigenous communities, social determinants of health

## Abstract

The objective of this review is to document contemporary barriers to accessing healthcare faced by Indigenous people of Canada and approaches taken to mitigate these concerns. A narrative review of the literature was conducted. Barriers to healthcare access and mitigating strategies were aligned into three categories: proximal, intermediate, and distal barriers. Proximal barriers include geography, education attainment, and negative bias among healthcare professionals resulting in a lack of or inadequate immediate care in Indigenous communities. Intermediate barriers comprise of employment and income inequities and health education systems that are not accessible to Indigenous people. Distal barriers include colonialism, racism and social exclusion, resulting in limited involvement of Indigenous people in policy making and planning to address community healthcare needs. Several mitigation strategies initiated across Canada to address the inequitable health concerns include allocation of financial support for infrastructure development in Indigenous communities, increases in Indigenous education and employment, development of culturally sensitive education and medical systems and involvement of Indigenous communities and elders in the policy-making system. Indigenous people in Canada face systemic/policy barriers to equitable healthcare access. Addressing these barriers by strengthening services and building capacity within communities while integrating input from Indigenous communities is essential to improve accessibility.

## 1. Introduction

In Canada, universal coverage for healthcare services is a strategy for “improving the lives of all Canada’s People and to making this country’s population among the healthiest in the world…” [1]. The Canada Health Act’s primary objective “… is to protect, promote, and restore the physical and mental wellbeing of residents of Canada and to facilitate reasonable access to health services without financial or other barriers” [2] while embracing diversity, creating healthy and respectful environments, and reducing health inequities [1]. Notwithstanding this, healthcare delivery often fails to address social and health inequities and may be influenced by ethnicity, culture and physical appearance, leading to health disparities among specific populations [3]. A large gap exists in health status between the general Canadian and Indigenous populations. Only 44% of First Nations (FN) people living on-reserve report very good/excellent health versus ~60% of non-Indigenous people [4] and FN people have shorter life expectancy [5], as do Inuit [6]. The prevalence of at least one chronic disease such as diabetes, obesity or arthritis is 60% in Indigenous adults [5]. In Canada, “Indigenous” or “Aboriginal” designate the three peoples who are originally from North America: FN, Inuit, and Métis [7]. Indigenous peoples officially account for 4.9% of the Canadian population [8] but, particularly in certain urban centers, the numbers may be greatly underestimated [9]. Overall, the population has expanded by 42.5% since 2006 and will exceed 2.5 million in the next twenty years [8], which will exacerbate unaddressed health disparities. Acknowledging the significant health gap between Indigenous and non-Indigenous populations, the Government of Canada (GoC) has implemented the Non-Insured Health Benefits (NIHB) program for FN and Inuit (but not Métis) people to help cover the costs of medically necessary items and services that are otherwise not insured [10]. Provincial programs for Indigenous people, such as the FN health contracted services agencies in British Columbia (BC) [11] also exist but health disparities, such as poor healthcare services access, remain a serious issue [12]. Research supports that equity-oriented healthcare produces better mental and physical health outcomes and better quality of life [13]. In light of the limited and outdated compilations of Indigenous healthcare access issues, this review presents a discussion of the current barriers and measures taken to reduce these discrepancies. Such a review is vital to highlight ongoing or new obstacles in healthcare access and assist Indigenous and non-Indigenous leaders to create action-oriented strategies and tools to effectively resolve health inequities in Canada.

## 2. Materials and Methods 

The review questions were: (1) What are the barriers faced by the Indigenous communities in accessing healthcare in Canada? (2) What strategies have been implemented to resolve these barriers and are there any plans to implement any new strategies in the future? The concepts included were: Indigenous population of Canada; barriers to healthcare access, social determinants of health; healthcare programs and policies, interventions.

We searched multiple databases and grey literature using a documented search strategy (Supplemental Table S1). The following databases were searched: PubMed, MEDLINE, EMBASE, iPortal, ProQuest. The grey literature search was conducted using the Google search engine and Google Scholar. Web pages of relevant organizations including First Nations, Métis, and Inuit were explored: University of Alberta Subject guide [14], Health Canada [15], Canadian Institutes of Health Research [16], Statistics Canada [17].

**Figure 1:**
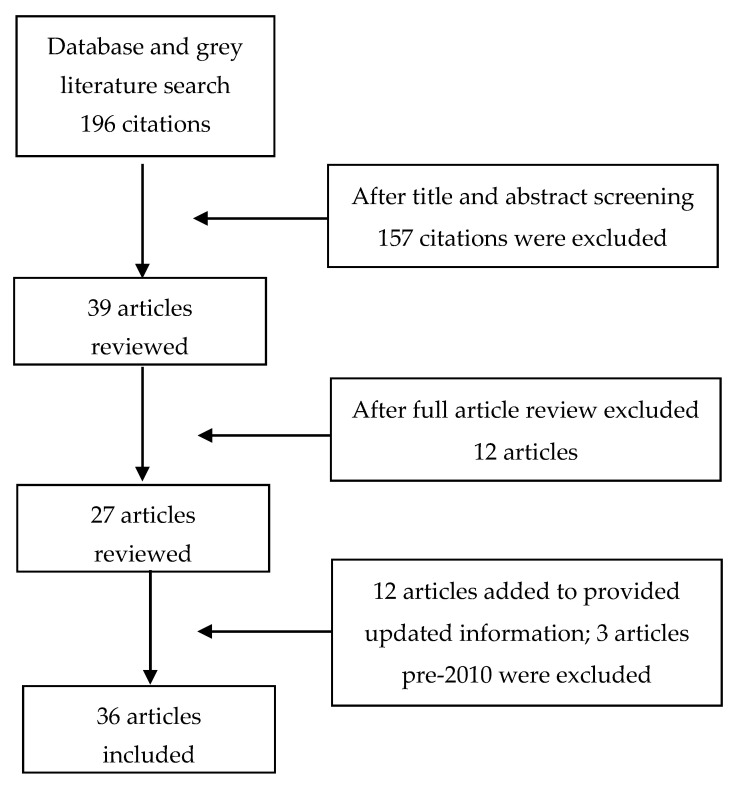
PRISMA flow diagram of articles included in the review.

A PICO framework was used. The population inclusion criteria were studies including adult Indigenous populations in Canada (First Nations, Metis, and Inuit) while the exclusion criteria were studies that did not specifically target indigenous populations but included indigenous participants. The interventions or exposures defined as studies in the English language, published between 2010 and May 2018, and describing the barriers to health care access in the Indigenous population in Canada were chosen (Figure 1). Interventions must have been applied to identify Indigenous people residing in Canada with the aim to improve access to healthcare and/or health outcomes. Types of studies included original research (prospective cohort, observational and qualitative studies), review articles, reports, policy documents and white papers. Statistical reports about the health status of the Indigenous communities were also included. Primary outcomes were common problems in healthcare access for Indigenous people and barriers to equitable healthcare access. Secondary outcomes were current and future strategies that have been or will be implemented to resolve the barriers. While OCAP^®^ has less applicability to secondary data analysis, the study team sought to be inclusive of Indigenous evaluation frameworks throughout this paper.

The initial search produced 196 articles from database searches and additional references from organization websites. After screening titles and reviewing abstracts, 157 articles were excluded. After a full review of 39 articles, a further 12 articles that did not fit the inclusion criteria were excluded, along with 3 that were older than 2010, leaving 25 articles that were included in the detailed analysis in this paper. In the final revision, 12 articles were added to provide updated information, thus a total of 36 articles were included (Supplementary Table S2).

## 3. Results

### 3.1. Data Extraction and Theming

Data extraction was carried out on the 36 selected articles and findings were categorized according to predetermined and emerging themes using a deductive-inductive approach based on the Structural and Social Determinants of Indigenous People’s Health Framework [18]. The main themes in the deductive framework included the following: (a) barriers faced by Indigenous people in accessing healthcare in Canada; (b) mitigation strategies implemented to resolve these barriers; (c) future plans to resolve these barriers to accessible healthcare. 

Following the Structural and Social Determinants of Indigenous People’s Health Framework, the findings of the review are summarized below according to the following themes: (1) proximal barriers to equitable healthcare access that affect individuals directly; (2) intermediate barriers to equitable healthcare access and (3) distal barriers to equitable healthcare access. Under each of the themes mentioned above, we identified sub-topics and mitigation strategies to resolve these barriers, described in detailed in the following sections. 

### 3.2. Proximal Barriers to Equitable Healthcare Access that Affect Individuals Directly 

Since these barriers can have a significant impact on many aspects of healthcare access, some of the proximal barriers could also be treated as intermediate barriers and vice versa.

#### 3.2.1. Geography

On remote and isolated FN reserves and other indigenous communities, health centers are mainly run by nurses or community health workers (CHW). Limitations on their scopes of practice, access to training and equipment required to treat residents in their community [19] means that FN people must travel to cities to access advanced treatment facilities and medical specialists, creating a geographical barrier to effective access [19,20] also pertinent to Métis and Inuit [21]. Many Inuit communities lack year-round road access and only a few have hospitals, hence travel by plane is required to reach the nearest hospital [21,22]. Weather is one factor that affects decisions to medically evacuate patients from remote locations and delays may severely affect the outcome of emergencies such as heart attacks [22]. Geography also limits high-speed internet infrastructure and access to online medical resources [23]. 

##### Mitigation Strategies

Recent initiatives to improve essential infrastructure include the Ontario provincial government partnering with three remote Northwestern Ontario FN communities to construct year-round road access [24] and the GoC’s 5-year, $1.5 billion investment to enhance access to critical medical care and services for Indigenous communities across Canada. The governments of Canada and Ontario collaborated to build new hospitals and ambulatory care facilities in remote Indigenous communities [10]. A plan to provide high-speed internet access to 190 Indigenous communities was developed [23]. Integration of telehealth provides greater access to specialized care and reduces travel and wait times by connecting patients from remote areas to healthcare providers (HCP) via teleconference [25]. Notable examples are the FN Telehealth Expansion Project in BC, telehealth services provided by Alberta Health and the Keewaytinook Okimakanak telemedicine program in northwestern Ontario [22,23,24,25,26,27,28]. Telehealth has successfully delivered many healthcare services to distant Indigenous communities [29], but cannot completely eradicate access barriers and requires ongoing maintenance of the infrastructure.

#### 3.2.2. Education Attainment 

Inadequate education often results in poor literacy, decreasing one’s ability to navigate the healthcare system [30]. A high proportion of Indigenous people still do not attend educational institutions [5,31], in part due to a lack of cultural sensitivity in education curricula [32]. For indigenous peoples living off-reserve, education attainment along with income and occupational status are the most important predictors of good health [33]. Low education, or low English or French literacy, may engender feelings of fear and intimidation when talking to healthcare providers (HCP) [34], creating a severe barrier because doctors may not refer patients to appropriate treatment if the health problem is not communicated and understood [35]. Due to language barriers and isolation from their communities, Indigenous people may refrain from traveling for health services [34].

##### Mitigation Strategies

Increasing access to education and improving the quality of curricula are essential to Indigenous students obtaining higher credentials. Collaborations between Indigenous communities, Indigenous and Northern Affairs Canada (INAC), the Assembly of FN and other organizations aim to improve education for FN children. An example is the Manitoba FN School System, the first program in Canada led by FN communities to ensure that elementary and secondary education programs are culturally relevant and high quality [36]. In 2016 the GoC funded $30 million to the Martin Family Initiative to improve elementary school educational outcomes in 20 schools in Ontario [37,38]. Financial support programs assisting Indigenous students to access education include the Indigenous Services Canada programs to Treaty and Status FN post-secondary students; “Indspire”, a national Indigenous registered charity organization, offers numerous scholarships and bursaries to Indigenous students pursuing post-secondary education, apprenticeships, skilled trades or technology programs [39]. See also the mitigation strategies section in Section 3.3.2 Health Education Systems for strategies specifically targeting healthcare training.

To facilitate communications between patients and HCP, Indigenous interpreters trained in medical terminology [35], such as the Aboriginal Patient Navigator (APN) program, are an important bridge. APNs assist patients in navigating the healthcare system and understanding medical advice and is available in various cities in BC, Newfoundland and Labrador, and Ontario [40,41,42].

#### 3.2.3. Negative Bias 

Colonization and historical intergenerational traumas (e.g., residential schools, Sixties Scoop) have plagued the survivors and later generations with physical and mental trauma, which can generate self-destructive behaviors such as alcoholism and violence [43]. These behaviors are often portrayed inaccurately in the media, leading to negative stereotypes of Indigenous people. For instance, some HCPs assume that all Indigenous people are addicts or alcoholic and that they are pretending to be sick in order to abuse medicine [4,44]. Therefore, physicians may limit interaction with these patients, offering them less chance to explain themselves [44] and possibly denying them specialist referrals [4]. Healthcare education systems offer limited cultural competency and sensitivity training, undermining non-indigenous HCPs’ ability to engage in meaningful conversations without offending their patients [3]. Many HCPs do not acknowledge the impact of colonization on the Indigenous community and disregard the social determinants of health (SDOH) as explanations for illness [4]. Such attitudes result in disrespectful, culturally inappropriate health services [4,34], which deter Indigenous people from seeking healthcare.

##### Mitigation Strategies

The creation of multidisciplinary teams including Indigenous people can enhance cultural sensitivity in clinical practice, such as mental wellness teams developed nationally. Teams of elders, social workers, nurses, psychologists and other experts develop and provide culturally appropriate and accessible mental health services for Indigenous communities [45]. The FN and Inuit Health Branch, the Assembly of FN and Indigenous mental wellness experts collaborated to create the FN Mental Wellness Continuum, which advises on policy and program changes that organizations can make to improve outcomes [45]. The Mental Health and Addiction Elders Advisory (MHAEA) in Alberta is a program created based on this continuum and is based on Indigenous knowledge, informed and guided by elders from Alberta FN communities [45]. A basic principle of Indigenous knowledge is recognizing the unique regional complexities of Indigenous communities [46]. Common elements of Indigenous knowledge include undertaking a wholistic health approach to wellness when reviewing experiences for critical analysis [47]. More strategies to avoid negative stereotypes of Indigenous patients are covered in the mitigation strategies section of Section 3.4.2 Racism and Social Exclusion.

#### 3.2.4. Insufficient Numbers or Retention of Qualified HCP

Despite the high prevalence of diseases and mortality, many indigenous communities lack sufficiently qualified HCP, thus patients may not get appropriate and timely diagnoses of more complex conditions [22]. A recent survey of 84 indigenous communities across Canada found that 58% of healthcare services were led by a nurse-in-charge versus 32% by physicians and 14% by nurse practitioners [48]. At Inuit community health centers, community health nurses are the backbone of the health system, providing most of the care through an expanded scope of practice [49]. In emergencies, lacking other resources, nurses often perform duties outside of their program-mandated scope of practice, such as prescribing medications or performing diagnostic imaging. Nurses must prioritize providing acute care, resulting in reduced emphasis on education and preventive services [50]. However, staff turnover rates are high, with incumbent disruptions in accessibility and excessive workload for those remaining [51]. In some communities, there are no full-time HCPs, meaning CHWs are the first responders. CHWs are lay residents of the communities and do not receive advanced clinical training; their intended role is to help in bridging cultural barriers between HCP and residents [52]. CHWs can be important for public health initiatives such as pediatric oral care [53] or chronic disease interventions [48], but privacy concerns of some community residents may also be a barrier [22].

##### Mitigation Strategies

Incentives such as scholarships, loan repayment programs, increased salary, and professional support programs can reduce staff turnover and increase recruitment into rural communities [54]. In 2013, the Government of BC and the BC Medical Association established “A GP for Me”, providing physicians committing to a 3-year contract in designated rural communities, including seven reserves, with financial incentives [55]. More than 3000 doctors participated, providing > 178,000 people with a family doctor [56]. The program laid a foundation for providing team-based care to meet patient needs and ongoing incentives for physicians for face-to-face interactions with patients and other specified activities [57]. 

Inefficient scheduling may exacerbate low staffing issues [22]. Thus, optimizing the scheduling process is necessary to maximize the presence of HCP, for example by balancing drop-in clinic times for urgent care with scheduled visits [58] and utilizing telehealth for follow-up with specialists [29]. 

To ensure the efficiency and effectiveness of healthcare services for Indigenous communities, it is essential to evaluate interventions [34]. The Canadian Institutes of Health Research (CIHR) recently launched the Pathways to Health Equity for Aboriginal People initiative that funds projects to design and promote programs and policies that support the well-being of Indigenous people [59].

### 3.3. Intermediate Barriers to Equitable Healthcare Access

#### 3.3.1. Employment and Income

The Canadian Community Well-Being Index takes into account community education, labor force participation, income and housing conditions. Although the overall Index rating of indigenous communities has increased over the last three decades, the gap between indigenous and non-indigenous communities has remained high [60]. In 2015, the unemployment rates of Indigenous versus general Canadian populations were 12.4% and 6.8%, respectively [61]. There is a significant wage gap with the median total income of FN individuals at $21,875 CAD compared with $34,604 CAD for non-Indigenous individuals [62]. This is lower than the low-income cut-off [63], below which families have no surplus to afford additional insurance or health services that are not covered by the NIHB or provincial or territorial governments [30]. This is a particular issue for diseases like diabetes that require supplies only partially covered by insurance [19]. Having a low income contributes to individuals not pursuing education and training [21,22] and also poses a barrier to travel for treatment off-reserve [64]. Although supplementary insurance coverage is provided to designated low-income residents and seniors, not all qualify for these benefits [65]. For instance, many Indigenous seniors cannot afford long-term care housing [64] and of the >600 FN across Canada, only 53 long-term care facilities are managed by FN, despite the growing demand. Restrictive wait-list policies may pose an additional barrier to seniors accessing off-reserve facilities [66].

##### Mitigation Strategies

In 2017–2018, INAC provided $4.6 billion over five years in support of affordable housing, travel expenses, education, gaining work experience and exploring different career options. INAC also invested $25 million over 5 years to develop a Métis Nation economic development strategy [67]. In the 2018 budget, the GoC proposed a 5-year, $2 billion investment for a new Indigenous skills and employment training program with an emphasis on higher paying jobs [10]. Provincial governments also support initiatives such as the Aboriginal Training to Employment Program in Alberta [68]. Various organizations have developed guides to help employers create inclusive and culturally sensitive workplaces [69,70], which in turn is expected to increase job satisfaction and retention of indigenous employees.

#### 3.3.2. Health Education Systems 

Post-secondary education in healthcare professions enables better employment and income for individuals and access to healthcare services for Indigenous populations. Significant socio-economic barriers and a history of discrimination and residential schools have resulted in poor educational attainment among Indigenous people. Furthermore, most current education curricula are not culturally competent; western medicine is preferred over traditional medicine (TM) and a lack of focus on traditional, holistic healthcare in the curriculum may discourage Indigenous students from pursuing healthcare careers [3,71].

##### Mitigation Strategies

Collaboration of Indigenous communities with universities is creating more equitable access to post-secondary education. Universities Canada, representing 95 universities, committed to enhancing education opportunities for Indigenous students and fostering reconciliation [72]. Currently, 70% of Canadian universities have partnerships with Indigenous communities; several are taking steps to include Indigenous representation in their governance and increase academic programs with an Indigenous focus [73]. Developing health education programs that put a greater emphasis on a holistic view of human health can motivate Indigenous youth to pursue health careers [74]. An excellent example is the University College of the North (UCN), Manitoba. To attract Indigenous students to its health sciences program, UCN offers cultural services, such as a counselling and elders’ programs, and provides a learning environment that allows students to honor and share their cultural beliefs and practices and promote cross-cultural awareness [75]. The UCN program includes courses focused on understanding the experiences of Indigenous communities in the past and present. All faculty and staff complete an Indigenous awareness course to promote culturally competence and sensitivity [75].

### 3.4. Distal Barriers to Equitable Healthcare Access

Distal barriers have the most profound influence on the healthcare access of Indigenous populations because these create intermediate and proximal barriers. Distal barriers can be the most difficult to change, yet, if transformed can significantly reduce health inequities and have the greatest health impacts [76].

#### 3.4.1. Colonialism 

Inequities towards Indigenous people in the Canadian healthcare system stem from the colonial foundations of Canada [71]. Colonialism created the political and economic inequalities that led to the abovementioned intermediate and proximal barriers to healthcare. One consequence of colonialism was the reserve system, which dispossessed many Indigenous peoples of their traditional lands and forced them to relocate to reserves in remote locations often lacking in critical infrastructure. Therefore, there are many challenges to establishing businesses or advanced healthcare facilities on reserves [77]. Colonialism blocked Indigenous people from engaging in policy making, which resulted in many decisions that continue to marginalize and exclude them and hinder their participation in decisions related to their healthcare priorities and needs [78], such as the under-development of necessary healthcare services on reserve areas, lack of culturally appropriate health services, underfunding and inappropriate health policies. For instance, many Indigenous people believe that TM is the most effective treatment for them, yet access is limited within healthcare settings oriented to western medicine [79]. However, incorporation of TM with western medicine is usually discouraged, and some physicians who would like to do so emphasize that incorporating alternative healing practices into the western medical system is challenging because the healthcare system is tightly controlled [4].

##### Mitigation Strategies

The reconciliation of colonialism’s impact requires collaboration between federal and provincial health systems and Indigenous communities to allow Indigenous representation on Regional Health Authorities and a permanent bilateral mechanism between governments and Indigenous people [25,34,35,64,80] and promotion of programs reflecting the community priorities [45]. A model of reconciliation and collaboration is the Toronto Indigenous Health Advisory Circle (TIHAC), a community-led advisory group in Ontario representing Indigenous people that advises on healthcare policy development [81]. The role of TIHAC is to guide identification, funding and evaluation of culturally based health services and influence public health policy that affects Indigenous health outcomes and addresses SDOH [81]. The TIHAC developed the Toronto Indigenous Health Strategy (TIHS) following the Indigenous medicine wheel and elders’ advice, and includes a wide range of strategies to improve healthcare accessibility. For instance: integrating more Indigenous navigators into the system, ensuring healthcare services are welcoming and inclusive of Indigenous people, providing education regarding Indigenous ceremonies/teachings, supporting health services that integrate TM with mainstream health programs and developing community-based healing/counseling teams of HCP [81]. 

Another example of Indigenous representation in a regulatory body is the formation of CIHR’s Indigenous People Health Institute Advisory Board, with seats reserved for delegates from the Assembly of FN, Inuit Tapiriit Kanatami and the Métis National Council [59]. CIHR holds meetings with senior leaders from Indigenous organizations across Canada to align research with community priorities [82] and including Indigenous communities in the policy discourse and policy-making process.

The federal government committed to transforming the health system into a model of self-determination, where services are developed, delivered and controlled by and for FN, including investment of $74.9 million to support a permanent bilateral mechanism to identify the communities’ priorities [10]. For example, the FN Health Authority (FNHA) in BC has a governance structure that includes representation from FN Health Council and FN Health Directors Association. FNHA has a provincial mandate to deliver programs and services previously provided by Health Canada. Through a tripartite governance framework between BC FN, the Province of BC and FNIHB, FNHA seeks to innovate and reform healthcare delivery by collaborating, coordinating and integrating health services and programs to address service gaps and achieve better health outcomes [83]. 

#### 3.4.2. Racism and Social Exclusion

Colonialism created social stratification, which led to inequitable distribution of resources, power, freedom and control among the Indigenous population, precipitating racism and social exclusion, impacting culturally sensitive access to the health system. Systemic racism (SR) and epistemic racism (ER) exist in healthcare [71]. SR refers to the “imposition and perpetuation of inequities through governance” [71]. For instance, NIHB referral forms require physicians to provide a patient’s personal information, regardless of their consent. If no information is received, the referral is denied. ER refers to “imposition of one world view over another” [71]; as discussed, the precedence of western medicine causes difficulties in accessing Indigenous TM, yet a convergence of traditional knowledge and western scientific methods has established the efficacy of traditional medicines in several instances [84]. Racism can also lead to unequitable resource allocation. For example, the sweat lodge is a traditional structure utilized by Indigenous people during certain ceremonies that is among the most commonly practiced cultural intervention and shown to provide benefit in conditions such as addictions [85]. Policies may favor allocating hospital funds to upgrade western medical technology instead of building sweat lodges [71]. 

Racism and social exclusion can also be observed in the practices of HCPs, including a lack of respect and inappropriate treatment [34]. Furthermore, inadequate health services offered on-reserve may prompt Indigenous people to relocate to cities, which can be terrifying as they often feel isolated and demoralized away from their home communities. These circumstances may prevent them from accessing or interacting with HCP [34].

##### Mitigation Strategies

Distal barriers necessitate that organizations review and revise their hiring policies and processes, performance evaluation, recognition and compensation to support the commitment to equitable health [25,67]. Practices include developing and delivering culturally sensitive healthcare services by: hiring Indigenous HCPs, or non-Indigenous staff who are motivated to work in Indigenous communities [25,58], developing anti-racism policies, and providing cross-cultural training [86]. Since 2016, Ontario requires mandatory cultural competency training for all public service employees including HCPs [87]; similarly, all existing and new Alberta Health Services staff are required to undergo Indigenous cultural competency training [88]. 

Another initiative that trains HCP and provides tools to eliminate healthcare inequities at the front line was developed by the interdisciplinary EQUIP team that includes researchers and knowledge users (HCPs and leaders in Indigenous health). It developed a series of tools and educational modules to help HCPs support equity-oriented practices and offer patient-centered care. The program examines SDOH and ways that clinics can support marginalized people. The tools support HCPs’ ability to speak to people without erroneous assumptions or unintentional negative bias [13]. 

Several services are offered across Canada to alleviate barriers to accessing high quality and culturally safe healthcare, such as the Aboriginal Patient Liaison programs and an Aboriginal Health Improvement Committee in northern British Columbia [89]. Indigenous Services Canada also supports national programs to improve Indigenous population health, such as the Aboriginal Diabetes Initiative and Fetal Alcohol Spectrum Disorder Program [90].

To prevent social exclusion, identification of opportunities to incorporate TM within hospital settings are necessary [30,35]. In rural northern Ontario, an Indigenous health framework and traditional healers are incorporated into the system, which has increased service accessibility and improved patient care [30].

## 4. Discussion

Indigenous people in Canada still face barriers to equitable healthcare access and more work is needed to eradicate this discrimination. Using the Social Determinants of Indigenous People’s Health Framework [1], we identified nine prominent barriers that contribute to difficulties in healthcare access in Indigenous communities. These barriers fall into three main categories: proximal, intermediate and distal. More specifically, proximal barriers include geography, education attainment, negative bias, and insufficient numbers of healthcare providers who may also have lower training qualifications than warranted by their responsibilities. Intermediate barriers consist of employment and income, and the lack of culturally relevant education system for healthcare professionals. Lastly, distal barriers include colonialism, and racism and social exclusion. All these barriers contribute to an intertwined and vicious cycle of difficulties in healthcare access in Indigenous communities.

The results from our review reiterate that the inequities towards Indigenous people in the Canadian healthcare system stem from the colonial foundations of Canada. Colonialism created the political and economic inequalities that led to the abovementioned intermediate and proximal barriers to healthcare. Similarly, two other review articles examining healthcare access issues in the Indigenous communities of Canada have stressed that the present healthcare disparities and inequitable health services are rooted in the past and present effects of colonialism [91,92]. The authors stated that the explanation for the health disparities extend beyond biomedical factors; rather, the evidence obtained from historical, social and political contexts explains the root causes of poor health and healthcare disparities in Indigenous communities [92]. 

We have presented the negative effects of colonialism that lead to social stratification, inequitable distribution of resources, power, freedom and control among the Indigenous peoples, precipitating racism and social exclusion, and disengagement of Indigenous people in policy making processes. Further negative bias and stereotypes and limited or no cultural sensitivity training for non-Indigenous healthcare staff has led to inadequate culturally sensitive services available to the Indigenous people. Using a postcolonial perspective, Horill et al. argued that racialized ideologies in the present-day healthcare system hold Indigenous people responsible for their own problems and poor health status [92]. Previous negative experience with the healthcare system was identified as a barrier to further interactions with the system, i.e., not seeking or delayed seeking of healthcare. These negative experiences also discourage trust in healthcare providers [92,93].

Our results identify that another implication of the colonial impact on healthcare acceptability is the importance of western medicine over traditional practices. Culturally safe and appropriate healthcare is an inter-related concept and involves the following key elements: healthcare services free of bias and discrimination; traditional and holistic healthcare needs of indigenous people; and active collaboration with Indigenous people as health providers [91]. We noted that the current education system lacks integration of holistic healthcare and traditional practices in the curricula, thus discouraging Indigenous students from pursing healthcare careers. This also limits the knowledge of traditional practices among the non-Indigenous students who will be future healthcare providers. Also, Indigenous people may not have trust in healthcare provided by government agencies due to the colonial history [92]. Hence, there is a need to include cultural and traditional practices in addition to or complementary to clinical healthcare [91]. Good communication between Indigenous people and their healthcare providers is key to regaining the acceptability of health services; this can be achieved by providing cultural safety training to healthcare staff as an integral part of healthcare institutions’ policy and programming [91]. Further trauma-informed compassionate care and working with Indigenous community members as active collaborative agents would help in the planning and implementation of patient-centered care [91].

More immediate factors such as geography, limited resources, limited healthcare staff, economic inequities and jurisdictional disputes on provision of care have been reported by us and others [91,92] as existing debilitating factors that limit healthcare availability to Indigenous people in Canada. Although these challenges impact the accessibility across all geographic locations, rural, remote and northern communities are the most severely affected by these factors [91]. 

Moving forward to ensure equitable healthcare services to Indigenous people, healthcare access should not be viewed as an individual or their community’s responsibility but instead considered as a social responsibility [92]. The Truth and Reconciliation Commission’s Call to Action #19 urges “the federal government, in consultation with Aboriginal peoples, to establish measurable goals to identify and close the gaps in health outcomes between Aboriginal and non-Aboriginal communities” [94]. The following mitigation strategies have been identified by other researchers to resolve these disparities: creating socially accepting and safe spaces, creating cultural safe education, research and healthcare practices [92,93], policy changes involving different levels of government to end jurisdictional disputes and provision of equitable funding [91,92], encouraging Indigenous people to develop healthcare policies and practices that are meaningful to them, and developing strategies for the recruitment and retention of healthcare staff to work in rural, remote and northern communities [91].

The mitigation strategies we have identified in this review resonate with the abovementioned suggestions and encompass changes in the provision of financial aid for infrastructure development and rural medical job retention, increases in Indigenous education and employment, development of culturally sensitive education and medical systems and, perhaps most importantly, involvement of Indigenous communities and elders in the policymaking system. Strengthening existing programs and creating similar initiatives in other domains of the SDOH, such as food security and adequate housing, are required to end inequitable healthcare access. Many programs are underway at federal, provincial and regional levels to increase the representation of Indigenous people in various governing bodies and committees. Effective health equity strategies need more work to spread beyond the health system to fully address SDOH. Moreover, development and implementation plans are necessary to increase communication about successful services to the public to raise awareness and public engagement.

There are several strengths and limitations to this review. This report directly considers evidence about healthcare access issues faced by FN, Inuit and Métis populations in Canada. By structuring the barriers into proximal, intermediate and distal components, this review demonstrates the complexity of barriers to healthcare access and emphasizes the importance of a holistic understanding of problems experienced by Indigenous communities in Canada. Another strength is that, where possible, we have used sources of information provided by the Indigenous people of Canada, for example the First Nations Information Governance Centre, which created OCAP^®^ and the First Nations Data Centre. We have also surveyed grey literature, which has provided insights into many programs and initiatives that are attempting to mitigate barriers to healthcare, but which may not have been evaluated using a traditional academic lens. This may also be viewed as a limitation of the work, in that the efficacy of these interventions is unclear and where evaluation has been done, scientific validity may be lacking. We acknowledge that the use of non-peer-reviewed sources such as organization or government reports, policy documents and white papers is a limitation; however, these materials are sometimes the only source of information regarding specific programs, for example, and thus have been included. As a narrative review, the purpose was to address the research questions as a summary overview of the main barriers and mitigating strategies with examples but is not an exhaustive catalogue.

This review provides up-to-date information on barriers and specific mitigation strategies that have been implemented by compiling and analyzing a wide range of relevant and recent journal articles, government and other published documents. This will facilitate the uptake and application of the review’s findings in future research and policy development. This review has some limitations that need to be considered. Due to the scarcity of relevant and specific information, a wide range of articles, including different methodologies and participant samples and populations, were used. Hence, a narrative review approach was used to synthesize the information. There is little information on the evaluation and success of the mitigation strategies described. Further research on the efficacy of various mitigation strategies is necessary for the determination of best practices.

## 5. Conclusions and Recommendations

Indigenous people face significant barriers to healthcare access in present day Canada. Addressing a few barriers ad hoc will not resolve the health inequities or improve overall health status of the Indigenous population. Therefore, the initiation and establishment of multi-sectoral, cross-government reforms involving a variety of strategies are necessary. Healthcare providers and policy makers need to be aware of the various challenges faced by Indigenous people, the context leading to these impairments and the cyclic and compounding effects of various biomedical, social, cultural, financial and political factors affecting the health of indigenous people. Cultural training and involvement of collaborative active Indigenous partners at all stages of planning and implementation stages of mitigation strategies and policy changes are essential to bring relevant, meaningful and sustainable changes. Lastly, in order to ensure reconciliation, every step necessary should be taken to close the gaps and inequities in health outcomes between Indigenous and non-indigenous people in Canada. 

## Supplementary Materials

The following are available online at https://www.mdpi.com/2227-9032/8/2/112/s1, Supplementary Table S1: Search words used in search engines and databases, Supplementary Table S2: Articles included in the review.
