# Supplementary material for: Barriers and Mitigating Strategies to Healthcare Access in Indigenous Communities of Canada: A Narrative Review
Source: Healthcare (Basel). 2020 Apr 26;8(2):112. doi: 10.3390/healthcare8020112 (PMC7349010; doi:10.3390/healthcare8020112)
Supplement: Supplementary file 1 [file healthcare-08-00112-s001.pdf]

healthcare

MDPI

Supplementary Material

Table 1. Search words used in search engines and databases.

Search Engines / Databases	Search Words
EMBASE	• Health service* accessibility<br>• Healthcare disparit*<br>• Barrier* OR facilitator OR approachability OR appropriate* accessibility OR accessible OR availability OR affordability<br>• Social determinants of health OR socioeconomic factors OR social support OR culturally appropriate OR community participation OR financial support OR financial hardship
Google	• Health care access<br>• Health care need<br>• Health services needs and demand<br>• Health care availability<br>• Delivery of health care<br>• Health care distribution<br>• Quality of healthcare
Google scholar	• Health service* accessibility<br>• Healthcare disparit*<br>• Barrier* OR facilitator OR approachability OR appropriate* accessibility OR accessible OR availability OR affordability<br>• Social determinants of health OR socioeconomic factors OR social support OR culturally appropriate OR community participation OR financial support OR financial hardship
iPortal	• Health service* accessibility<br>• Healthcare disparit*<br>• Barrier* OR facilitator OR approachability OR appropriate* accessibility OR accessible OR availability OR affordability<br>• Social determinants of health OR socioeconomic factors OR social support OR culturally appropriate OR community participation OR financial support OR financial hardship
MEDLINE	• ("Improve*" OR "Increase*") ("health care access*" OR "health access*") for ("indigenous*" OR "aboriginal*" OR "First Nation*" OR "Inuit*" OR "Metis")<br>• (Improv* OR Increas* OR Enhanc* OR Promot*) AND ("health care access*" OR "health access*" OR "health") AND (indigenous OR native OR aboriginal OR indians OR first nations) AND (in Canada) AND ("strateg*" OR "solution*")
ProQuest	• Health service* accessibility<br>• Healthcare disparit*<br>• Barrier* OR facilitator OR approachability OR appropriate* accessibility OR accessible OR availability OR affordability<br>Social determinants of health OR socioeconomic factors OR social support OR culturally appropriate OR community participation OR financial support OR financial hardship
PubMed	• ("Improve*" OR "Increase*") ("health care access*" OR "health access*") for ("indigenous*" OR "aboriginal*" OR "First Nation*" OR "Inuit*" OR "Metis")<br>(Improv* OR Increas* OR Enhanc* OR Promot*) AND ("health care access*" OR "health access*" OR "health") AND (indigenous OR native OR aboriginal OR indians OR first nations) AND (in Canada) AND ("strateg*" OR "solution*")

Table 2. Articles included in the review.

Publication	Theme	Sub-topic	Mitigation Strategies
Beatty, B.B and Berdahl, L., 2011. [64]	Intermediate Barriers	Employment and income	
Beckett, M., et al., 2018. [86]	Intermediate Barriers		Health education systems; Racism and social exclusion
Browne, A.J. et al., 2016. [58]	Proximal Barriers		Insufficient numbers or retention of qualified HCP
Cameron, B.L. et al., 2014. [34]	Proximal and Distal Barriers	Education attainment; Negative bias; Racism and social exclusion	Insufficient numbers or retention of qualified HCP
Fridkin, A.J., 2012. [78]	Distal Barriers	Colonialism	
Government of Canada, 2018. Budget plan 2018 [10]	Proximal, Intermediate and Distal Barriers		Geography; education and employment; colonialism
Haddad, P.S. et al., 2012 [84]	Distal Barriers	Racism and social exclusion	
Indigenous and Northern Affairs Canada, 2017. [37]	Proximal Barriers		Education attainment
Institute for Circumpolar Health Research, 2016. [79]	Distal Barriers	Colonialism	
Interior Health Authority, 2019. [42]	Proximal Barriers		Education attainment
Inuit Tapiriit Kanatami, 2014. [21]	Proximal Barriers	Geography	
Island Health, 2016. [25]	Distal Barriers		Racism and social exclusion
Kulhawy-Wibe, S. et al. 2018 [20]	Proximal Barriers	Geography	
Lavoie, J.G., 2013. [80]	Distal Barriers		Colonialism
Lewis, M. and Myhra, L.L., 2017. [30]	Intermediate and Distal Barriers	Education attainment; Employment and income	Colonialism; Racism and social exclusion
Mathu-Muju, K.R. et al., 2017. [53]	Proximal Barriers	Insufficient numbers or retention of qualified HCP	
Matthews, R., 2017. [71]	Intermediate and Distal Barriers	Health education systems; Colonialism; Racism and social exclusion	
McCalman, J. et al., 2019. [51]	Proximal Barriers	Insufficient numbers or retention of qualified HCP	
McDonnell, L. et al., 2019. [49]	Proximal Barriers	Insufficient numbers or retention of qualified HCP	
Misfeldt, R. et. al., 2013. [54]	Proximal Barriers		Insufficient numbers or retention of qualified HCP
Nader et al., 2017. [3]	Proximal Barriers	Negative Bias	
National Collaborating Centre for Aboriginal Health, 2017. [61]	Intermediate Barriers	Employment and income	
Nelson, E., 2017. [19]	Proximal Barriers	Geography	

O'Neil, J., et al., 2016 [83]	Distal Barriers		Colonialism
Oosterveer, T.M., and Young, T.K., 2015. [22]	Proximal Barriers	Geography; Insufficient numbers or retention of qualified HCP	
Reading, C.L., and Wien, F., 2009. [18]	Social Determinants of Indigenous People's Health Framework		
Restoule, B.M. et. al., 2016. [45]	Proximal and Distal Barriers		Negative bias; Colonialism
Rowan, M. et al., 2014. [85]	Distal Barriers	Racism and social exclusion	
Saskatchewan Health Authority, 2010. [35]	Proximal and Distal Barriers	Education attainment	Education attainment; Colonialism; Racism and social exclusion
Tang, S.Y. et. al., 2015. [44]	Proximal Barriers	Negative Bias	
The National Indigenous Economic Development Board, 2019. [77]	Distal Barriers	Colonialism	
Toronto Indigenous Health Advisory Circle, 2016. [81]	Distal Barriers		Colonialism
Toulouse, J., 2016. [74]	Intermediate Barriers		Health education systems
Wilk, P. et al., 2017. [43]	Proximal Barriers	Negative bias	
Wylie, L. and McConkey, S., 2018. [4]	Proximal Barriers	Negative Bias	
Zeran,V., 2016. [75]	Intermediate Barriers		Health education systems
